# Mechanistic investigation of the formation of H_2_ from HCOOH with a dinuclear Ru model complex for formate hydrogen lyase

**DOI:** 10.1080/14686996.2017.1379857

**Published:** 2017-11-01

**Authors:** Taisuke Tokunaga, Takeshi Yatabe, Takahiro Matsumoto, Tatsuya Ando, Ki-Seok Yoon, Seiji Ogo

**Affiliations:** ^a^ International Institute for Carbon-Neutral Energy Research (WPI-I2CNER), Kyushu University, Fukuoka, Japan; ^b^ Center for Small Molecule Energy, Kyushu University, Fukuoka, Japan; ^c^ Department of Chemistry and Biochemistry, Graduate School of Engineering, Kyushu University, Fukuoka, Japan

**Keywords:** Mechanistic investigation, H_2_ evolution, formic acid, formate hydrogen lyase model, dinuclear Ru complex, 30 Bio-inspired and biomedical materials, 106 Metallic materials

## Abstract

We report the mechanistic investigation of catalytic H_2_ evolution from formic acid in water using a formate-bridged dinuclear Ru complex as a formate hydrogen lyase model. The mechanistic study is based on isotope-labeling experiments involving hydrogen isotope exchange reaction.

## Graphical Abstract

**Figure:**
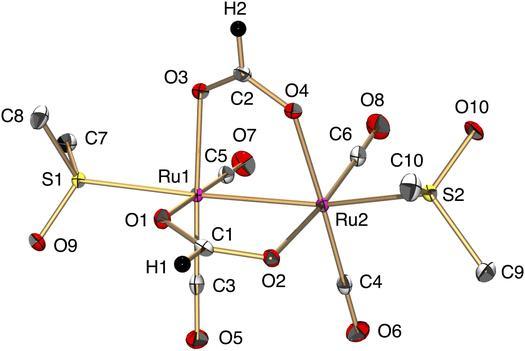

## 1. Introduction

Formate hydrogen lyase (FHL) is an enzyme complex that catalyzes the conversion of HCOOH to evolve CO_2_ and H_2_ (Eq. 1) [1–3]. This enzyme complex is composed of a formate dehydrogenase (FDH) [4,5] and a [NiFe]hydrogenase ([NiFe]H_2_ase) [6–9], and catalyzes oxidation of HCOOH (Eq. 2) and evolution of H_2_, respectively (Eq. 3).(1)
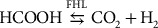

(2)
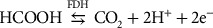

(3)
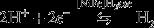



We have previously reported a Ni^II^Ru^II^ model complex [Ni^II^(X)(HCOO)(μ-H)Ru^II^(C_6_Me_6_)] (X = *N*,*N*′-dimethyl-3,7-diazanonane-1,9-dithiolato) that can catalyze fast conversion of HCOOH to H_2_ and CO_2_ [10] [turnover frequency {TOF = (mol of evolved H_2_/mol of catalyst) per hour} = 857 h^−1^]. This extremely fast reaction rate, however, prevented us from investigating the mechanism of H_2_ formation.

As part of our efforts to investigate the mechanism of H_2_/CO_2_ formation from HCOOH, we now report a dinuclear Ru^I^ complex [Ru^I^_2_(CO)_4_(μ-HCOO)_2_(DMSO)_2_] (**1**) that catalyzes the above reaction at a significantly slower reaction rate (TOF = 13.1 h^−1^). Here we disclose the detailed mechanism of H_2_ formation from HCOOH based on isotope-labeling experiments.

## 2. Experimental details

## 2.1. Materials and methods

All experiments were carried out under N_2_ or Ar atmosphere by using standard Schlenk techniques and a glovebox. Tetrahydrofuran (THF) was distilled from Na/benzophenone under N_2_ atmosphere prior to use. HCOOH, DCOOD, 40% NaOD/D_2_O, and dimethylsulfoxide (DMSO) were purchased from Wako Pure Chemical Industries, Ltd. (Osaka, Japan). HCOOD and DCOOH were purchased from Tokyo Chemical Industry Co., Ltd. (Tokyo, Japan). These materials were used without further purification. Proton nuclear magnetic resonance (^1^H NMR) spectra were recorded on a JEOL JNM-AL300 spectrometer (JEOL, Tokyo, Japan) at 25 °C, in which the chemical shifts were referenced to tetramethylsilane (TMS) in chloroform-*d*_1_ and DMSO in DMSO-*d*_6_. UV–vis absorption spectra were recorded on a JASCO V–670 UV–Visible–NIR spectrophotometer (the light pass length was 1.0 cm). An IR spectrum was recorded on a Thermo Nicolet NEXUS 870 Fourier transform infrared (FTIR) instrument (Thermo Fisher Scientific, Massachusetts, USA) at 25 °C. Gas chromatographic (GC) analyses were conducted by a Shimadzu GC–8A (He carrier) (Shimadzu, Kyoto, Japan) with a MnCl_2_–alumina column (model: Shinwa OGO–SP) at –196 °C (liquid N_2_) for quantitative analyses of H_2_, HD, and D_2_ and by a Shimadzu GC-2014 (Ar carrier) (Shimadzu, Kyoto, Japan) with activated charcoal at 100 °C for quantitative analyses for H_2_, HD, D_2_, and CO_2_ using a thermal conductivity detector. Elemental analysis data were obtained by a PerkinElmer 2400II series CHNS/O analyzer (PerkinElmer, Massachusetts, USA) using Ar as the carrier gas. Dynamic light scattering measurements were conducted with a Malvern Zetasizer Nano (Malvern, Worcestershire, UK). X-band electron spin resonance (ESR) spectra were measured by a JEOL JES-FA200 spectrometer (JEOL, Tokyo, Japan) at –150 °C.

**pH Adjustment.** The pH of the solution was adjusted by using HCOOH and NaOH/H_2_O (1.0–7.0). The pD of the solution was adjusted by using HCOOH, DCOOH, HCOOD, DCOOD, and 40% NaOD/D_2_O (1.0–7.0), in which H^+^ concentration of HCOOH and DCOOH is negligible quantity compared to D^+^ concentration of D_2_O. In a pH (or pD) range of 1.0–7.0, the pH (or pD) values of the solutions were determined by a pH meter (model: TOA HM20 J; DKK-TOA, Tokyo, Japan) equipped with a pH combination electrode (model: TOA GST-5725C; DKK-TOA, Tokyo, Japan). Values of pD were corrected by adding 0.4 to the observed values (pD = pH meter reading + 0.4) [11,12].

**[Ru** ^**I**^_**2**_ **(CO)**_**4**_ **(μ-HCOO)**_**2**_ **(DMSO)**_**2**_ **] (1).** A THF solution of [Ru^0^_3_(CO)_12_] (1.00 g, 1.56 mmol) and HCOOH (12 mL, 0.32 mol) was refluxed for 3.5 h. The resulting solution was cooled to room temperature, to which was added DMSO (335 μL, 4.72 mmol) followed by its stirring for 30 min. The solvent was removed under reduced pressure to yield a yellow powder of **1**, which was collected and dried in vacuo {yield: 85% based on the amount of [Ru^0^_3_(CO)_12_] added}. ^1^H NMR (300 MHz, in chloroform-*d*_1_, referenced to TMS): *δ* 8.23 (s, 2H, *H*COO), 3.06 (s, 12H, (C*H*_3_)_2_SO). Anal. Calcd for **1** (C_10_H_14_O_10_Ru_2_S_2_): C, 21.43; H, 2.52%. Found: C, 21.45; H, 2.22%.

**Typical procedure of H**_**2**_ **evolution from HCOOH catalyzed by 1.** In a 3.0 mL vial capped with a septum, a solution of HCOOH (2.60 mmol) in H_2_O (1.0 mL) was added to **1** (1.25 μmol). The pH of the resulting solution was adjusted to 1.0–7.0 and the solution was heated at 80 °C for 1 h. The gas above the solution within the vial was sampled with a gas-tight syringe (500 μL) and analyzed for H_2_ and CO_2_ by GC. No CO was observed. No nanoparticles were formed in the catalytic reaction, which was confirmed by dynamic light scattering measurements.

**Isotope-labeling experiments for catalytic H**_**2**_ **, HD, and D**_**2**_ **evolution.** In a 3.0 mL vial capped with a septum, DCOOH (2.60 mmol) in H_2_O (1.0 mL), HCOOD (2.60 mmol) in D_2_O (1.0 mL), or DCOOD (2.60 mmol) in D_2_O (1.0 mL) was added to **1** (1.25 μmol). The pH or pD of the resulting solution was adjusted to 3.5 by the addition of NaOH or NaOD, respectively, and it was stirred at 80 °C for 1 h. The gas above the solution within vial was sampled with a gas-tight syringe (500 μL) and analyzed for H_2_, HD, D_2_, and CO_2_ by GC. No CO was observed. No nanoparticles were formed in the catalytic reaction, which was confirmed by a dynamic light scattering measurements.

**The initial rate of the catalytic H**_**2**_ **evolution against the catalyst concentration.** In a 3.0 mL vial capped with a septum, a solution of HCOOH (2.60 M) in H_2_O was added into **1** (0.63, 1.25, 2.5, or 5.0 mM). The pH of the resulting solution was adjusted to 3.5 and the solution was heated at 80 °C for 300 s. The gas above the solution within the vial was sampled with a gas-tight syringe (500 μL) and analyzed for H_2_ by GC. The catalytic reaction is first-order against the catalyst concentration.

**Reactivity of hydride species 2 toward proton of HCOOH.** In a 3.0 mL vial capped with a septum, 10 equivalents of HCOOH (9.4 μL, 250 μmol) was added into a DMSO (2.0 mL) solution of **2** (vide infra), which was prepared from the reaction of **1** (14 mg, 25 μmol) with HCOONa (1.7 mg, 25 μmol) at 80 °C for 1 h. No H_2_ gas was formed, as confirmed by GC analysis. The same reaction was conducted using DMSO-*d*_6_ (450 μL) instead of DMSO (2.0 mL), which was monitored by ^1^H NMR spectroscopy. No decrease of the hydride-derived peak of **2** was observed.

**H** ^**+**^ **/D** ^**+**^ **exchange of hydride ligand of 2.** In an NMR sample tube, a DMSO-*d*_6_ solution (400 μL) of **1** (14 mg, 25 μmol) with HCOONa (1.7 mg, 25 μmol) was heated at 80 °C for 1 h to form **2**, which was confirmed by ^1^H NMR spectroscopy. Then, D_2_O (50 μL) was added into the resulting DMSO-*d*_6_ solution under N_2_ atmosphere. The H^+^/D^+^ exchange of hydride ligand of **2** was confirmed by ^1^H NMR spectroscopy with CH_2_Br_2_ as an internal standard to investigate the intensities of hydride- and formate-derived peaks.

**X-ray crystallographic analysis of 1.** A single crystal of **1** suitable for X–ray analysis was obtained from the diffusion of diethyl ether into its THF solution. Measurements were performed on a Rigaku/MSC Saturn CCD diffractometer (Rigaku, Tokyo, Japan) with confocal monochromated Mo-K*α* radiation (*λ* = 0.7107 Å). Data were collected and processed using the CrystalClear program. All calculations were performed using the CrystalStructure crystallographic software package except for refinement, which was performed using SHELXL–97. Crystallographic data for **1** have been deposited at the Cambridge Crystallographic Data Centre as Supplementary Publication No. CCDC 1556459. Copies of the data can be obtained free of charge on application to CCDC, 12 Union Road, Cambridge CB2 1EZ, UK {fax.: (+44)1223–336–033; e-mail: deposit@ccdc.cam.ac.uk}.

## 3. Results and discussion

**Figure 1.**
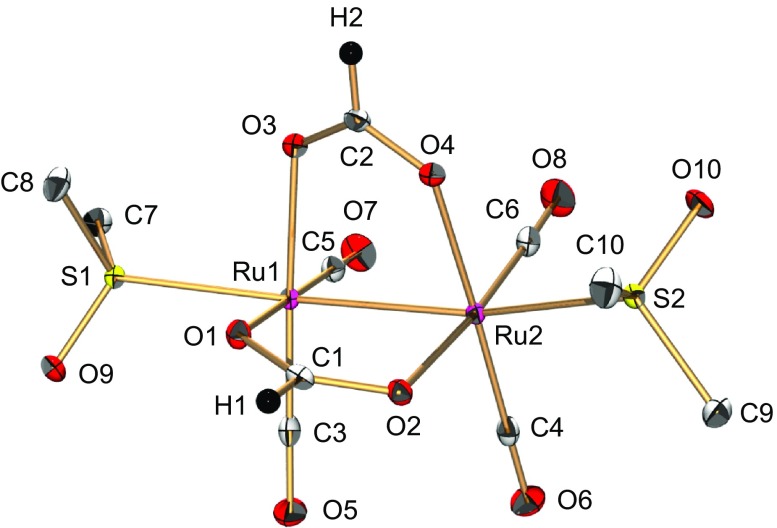
An ORTEP drawing of **1** with thermal ellipsoids at 50% probability. The hydrogen atoms of DMSO are omitted for clarity. Selected interatomic distances (*l*/Å) and angles (*ϕ*/°): Ru1–Ru2 = 2.6654(3), Ru1–O1 = 2.1152(13), Ru1–O3 = 2.1290(13), Ru1–S1 = 2.4107(5), Ru1–C3 = 1.855(2), Ru1–C5 = 1.853(2), Ru2–O2 = 2.1374(13), Ru2–O4 = 2.1221(13), Ru2–S2 = 2.4134(5), Ru2–C4 = 1.855(2), Ru2–C6 = 1.849(2), C1–O1 = 1.258(2), C1–O2 = 1.262(2), C2–O3 = 1.263(2), C2–O4 = 1.259(2), C3–O5 = 1.145(2), C4–O6 = 1.142(2), C5–O7 = 1.148(2), C6–O8 = 1.146(2), O1–C1–O2 = 126.67(18), O3–C2–O4 = 126.76(17).

**Figure 2.**
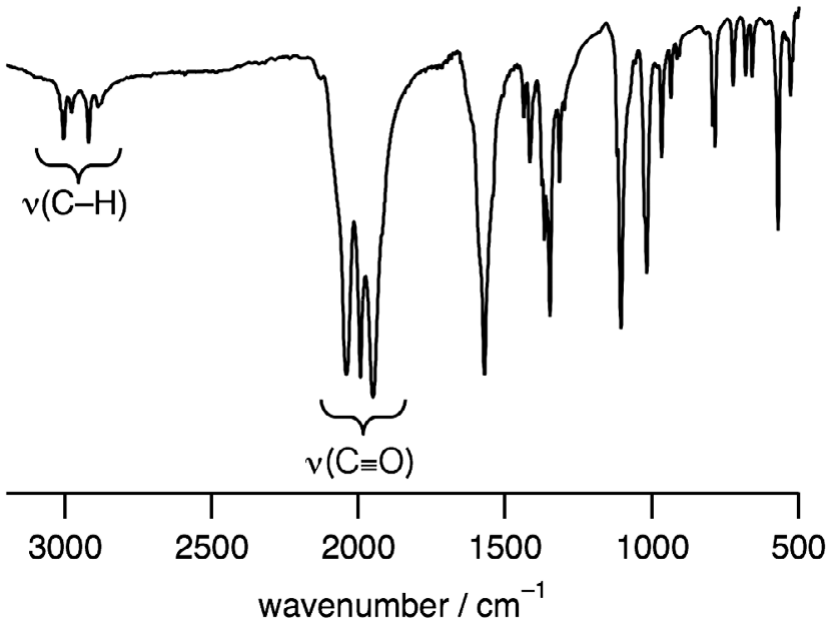
An IR spectrum of **1** in a KBr disk.

**Figure 3.**
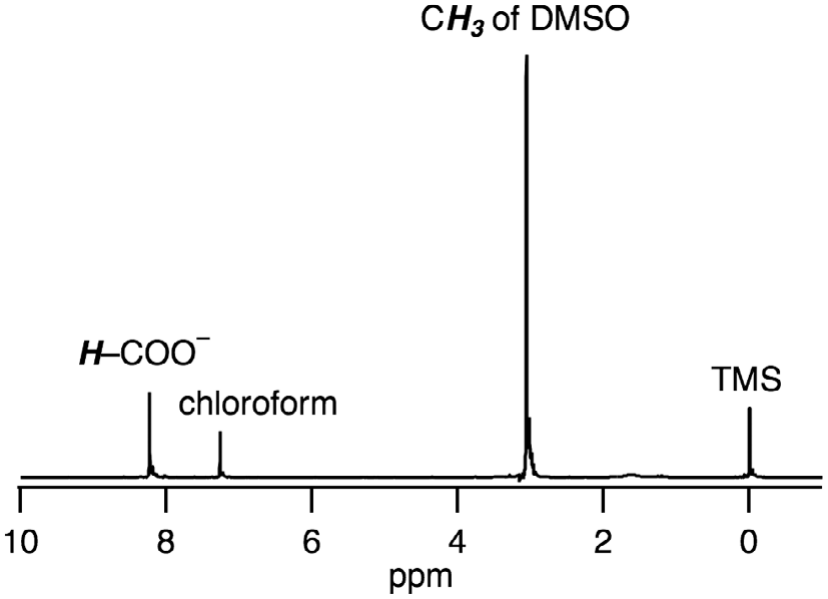
A ^1^H NMR spectrum of **1** in chloroform-*d*_1_.

**Figure 4.**
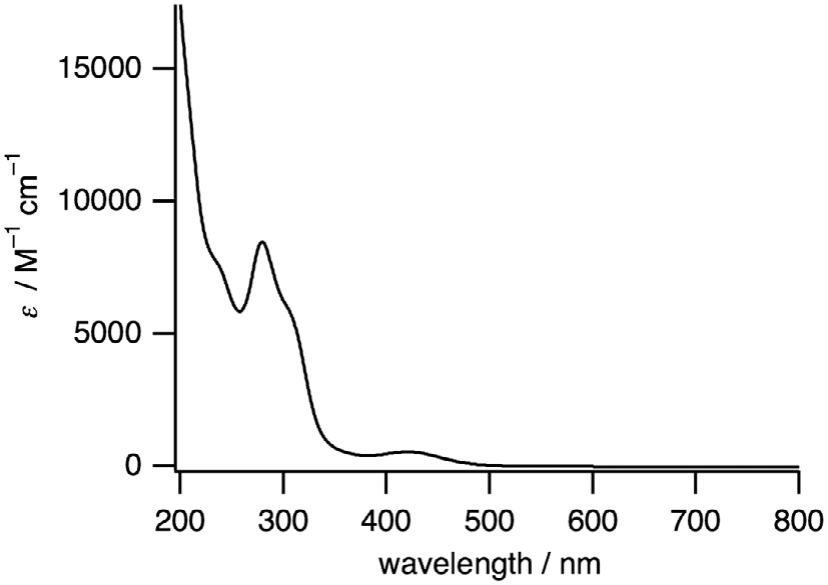
A UV-vis absorption spectrum of **1** (0.060 mM) in H_2_O.

A bis(μ-formate) Ru^I^_2_ complex, [Ru^I^_2_(CO)_4_(μ-HCOO)_2_(DMSO)_2_] (**1**) was synthesized from the reaction of [Ru^0^_3_(CO)_12_] with HCOOH and DMSO in THF under a N_2_ atmosphere, which was then characterized by X-ray analysis, and ^1^H NMR, UV-vis, and IR spectroscopies (Figures 1–4). Both Ru atoms adopt a distorted octahedral geometry in which the Ru ion is ligated by two C(CO), an S(DMSO), two O(μ-formate), and an adjacent Ru atom (Figure 1). Two Ru atoms are tethered by a Ru–Ru bond and two formate ligands. The bond distance of Ru1–Ru2 {2.6654(3) Å} is similar to those of other formate-bridged dinuclear Ru complexes (2.679 and 2.720 Å) [13,14]. The C–O bond distances of each HCOO^–^ ligand {C1–O1 = 1.258(2), C1–O2 = 1.262(2), C2–O3 = 1.263(2), and C2–O4 = 1.259(2) Å} are almost the same, which indicates that this could be explained by the delocalization of a double bond over two C–O bonds. The C–O bond distances of four CO ligands {1.142(2)–1.148(2) Å} are slightly longer than that of free CO (1.128 Å), suggestive of back donation of electron density from the *t*_2 g_ orbital of low-valent Ru^I^ to the π*-antibonding orbital of CO.

The IR spectrum of **1** shows the stretching frequencies of CO coordinated to Ru^I^ centers at 1950, 1994, and 2041 cm^−1^ (Figure 2), which are lower than the free CO stretching frequency (2143 cm^−1^). This weakened CO bond is also caused by the back donation from the low-valent Ru^I^ to the CO ligand. The ^1^H NMR signals observed in the diamagnetic region revealed that **1** is diamagnetic, which originates from an antiferromagnetic exchange interaction between two Ru^I^ centers through metal–metal bonding (Figure 3). The UV-vis absorption spectrum of **1** in H_2_O shows a sharp band at 280 nm (8480 M^−1^ cm^−1^) and a broad band at 420 nm (550 M^−1^ cm^−1^) (Figure 4).

**Figure 5.**
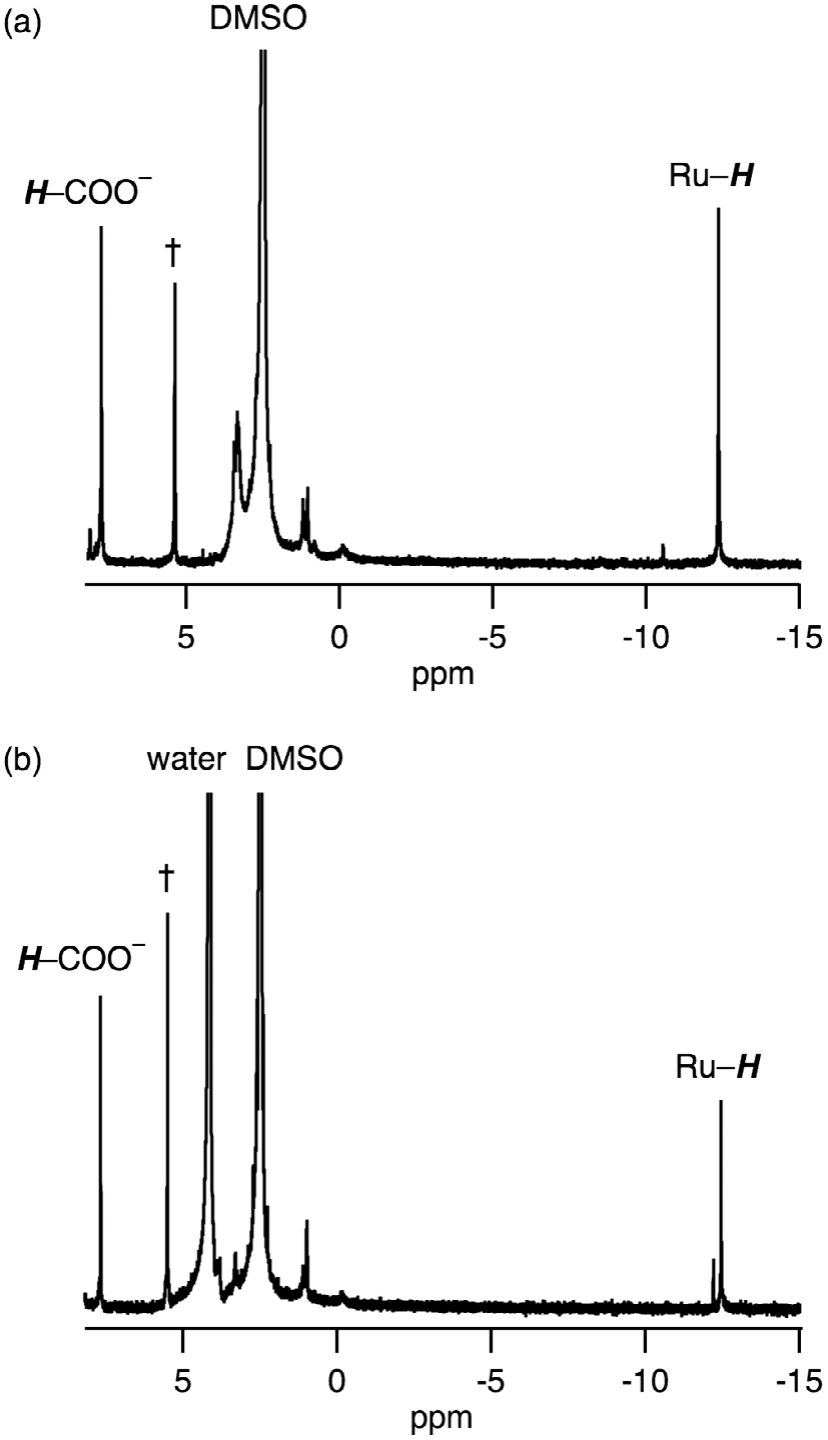
^1^H NMR spectra of **2** (a) before and (b) after addition of D_2_O, in which **2** was obtained from heating of **1** at 80 °C in DMSO-*d*_6_ for 1 h in the presence of 1 equivalent of HCOONa. †: the peak of CH_2_Br_2_ as an internal standard.

The bis(μ-formate) Ru^I^_2_ complex **1** can convert to a (hydride)(formate) species **2** with evolution of CO_2_ in DMSO at 80 °C in the presence of 1 equivalent of HCOONa. β-Hydrogen elimination is expected to be assisted by the metal centers to release CO_2_ and form the hydride ligand [15]. The ^1^H NMR spectrum of **2** in DMSO-*d*_6_ shows hydride- and formate-derived signals at –12.4 and 7.76 ppm, respectively, with the same intensities (Figure 5(a)). The hydride-derived peak is typical of those found in hydride-bridged Ru complexes [16–19]. The dimer structure of **2** with an antiferromagnetic exchange interaction between two Ru^I^ centers, was suggested by the signals observed in the diamagnetic region of the ^1^H NMR spectrum and its ESR silent character.

We have confirmed that the hydride ligand of **2** has a protic character rather than hydridic character based on the following investigations. We observed **2** being unreactive toward proton of HCOOH, i.e. dihydrogen gas was not formed via protonation of the hydride ligand, which was confirmed by ^1^H NMR spectroscopy and GC analysis. Then, we observed an H^+^/D^+^ exchange of the hydride ligand of **2**, as confirmed by ^1^H NMR spectroscopy (Figure 5). The intensity of hydride-derived peak of **2** only decreased by the addition of D_2_O into DMSO-*d*_6_ solution of **2** (Figure 5), meaning that the hydride ligand underwent the H^+^/D^+^ exchange with D^+^.

**Figure 6.**
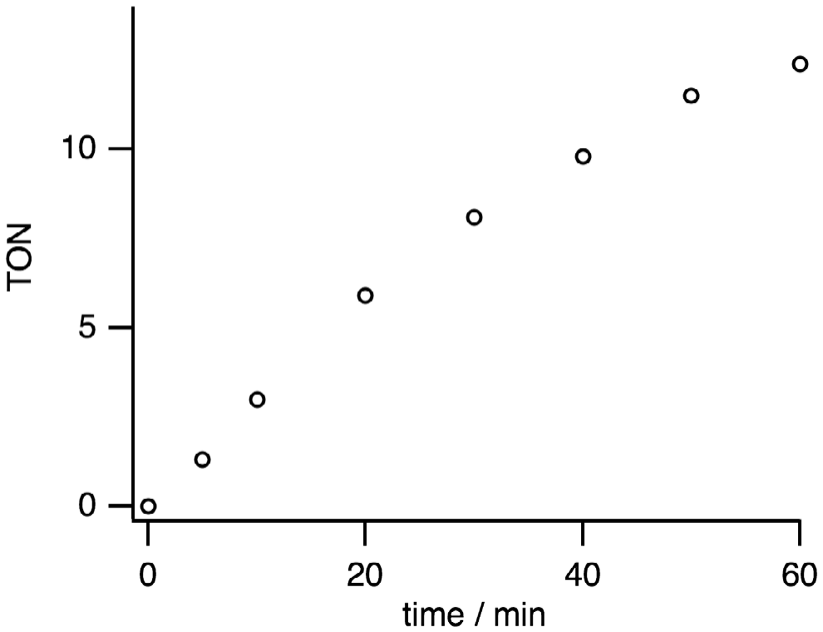
A plot of turnover numbers (TONs) vs. time for the H_2_ evolution from HCOOH catalyzed by **1** (1.25 mM) with HCOOH (2.60 M) in H_2_O at 80 °C and pH 3.5.

**Figure 7.**
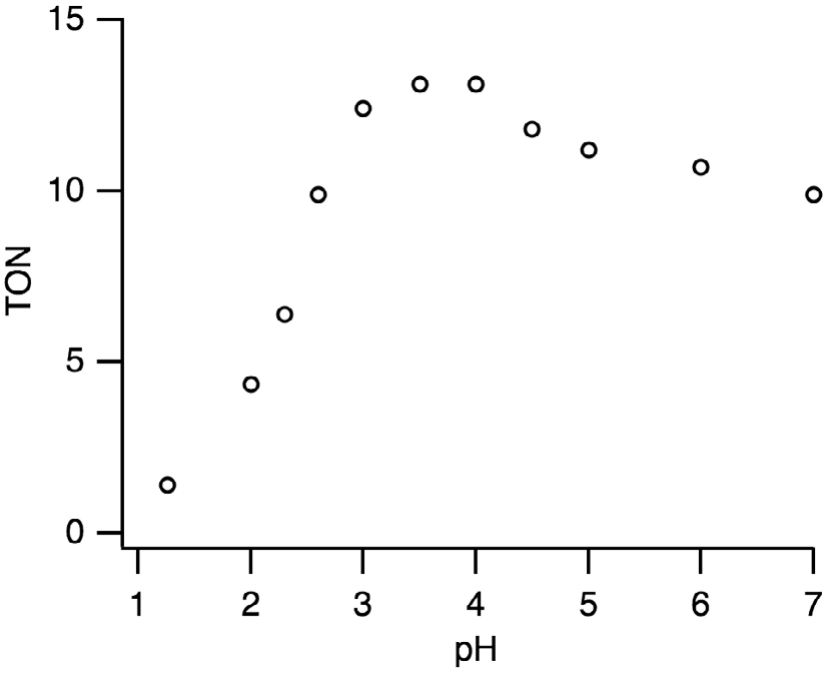
A pH-dependent H_2_ evolution from HCOOH catalyzed by **1** (1.25 mM) with HCOOH (2.60 M) in H_2_O for 1 h at 80 °C. The maximum turnover number (TON) is 13.1 at pH 3.5.

**Figure 8.**
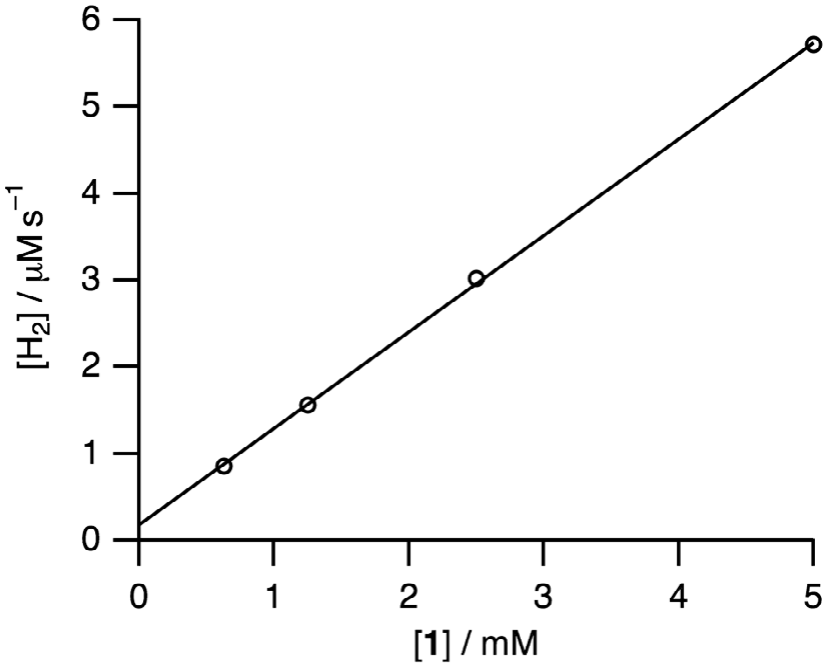
A plot of initial rate of H_2_ production against the concentration of **1** (0.63–5.0 mM) in the reaction of **1** with HCOOH (2.60 M) at 80 °C and pH 3.5. The initial rate of H_2_ production was determined for 300 s.

**Figure 9.**
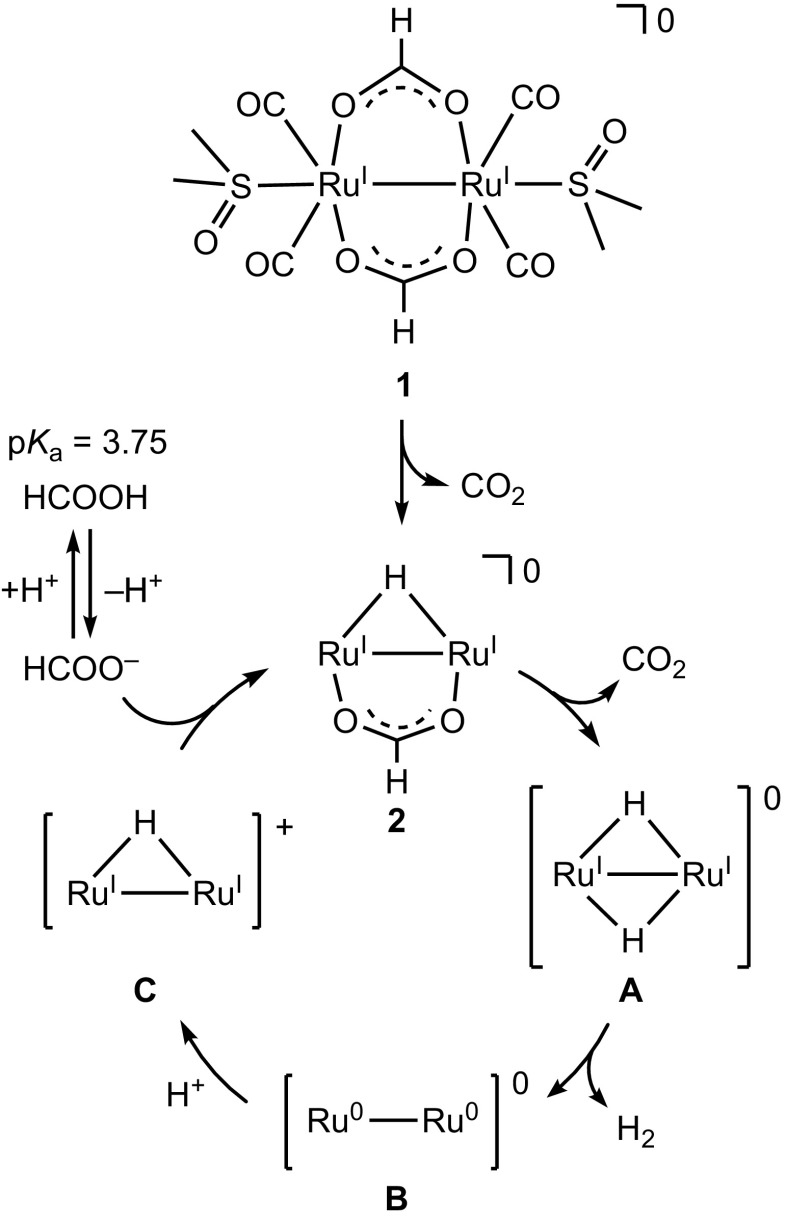
A proposed reaction mechanism for the conversion of HCOOH to H_2_ and CO_2_ catalyzed by dinuclear Ru complexes in H_2_O.

**Figure 10.**
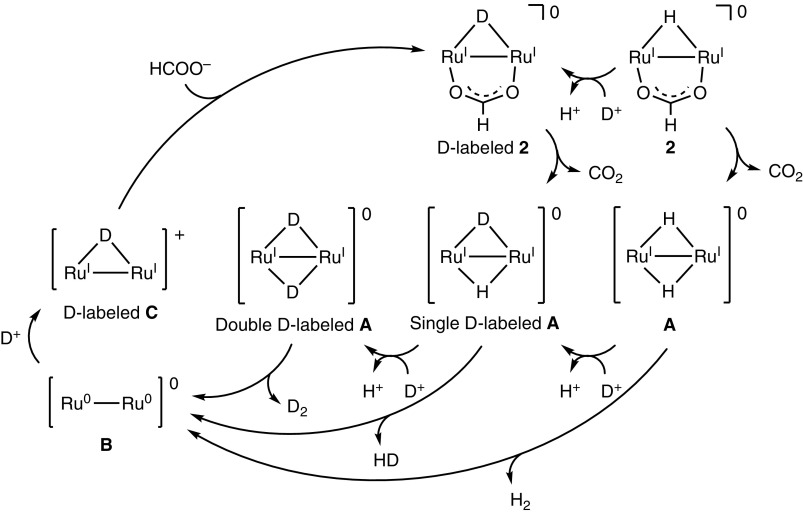
A proposed reaction mechanism for the hydrogen isotope exchange reaction in the conversion of HCOOD to H_2_, HD, D_2_, and CO_2_ in D_2_O catalyzed by dinuclear Ru complexes (entry 3 of Table 1).

Complex **1** is a precursor to catalyze the pH-dependent conversion of HCOOH to H_2_ and CO_2_ in water at pH 1.0–7.0 (Figures 6–10 and Table 1). H_2_ and CO_2_ gases were detected by GC. No nanoparticles were formed in the catalytic reaction, which was confirmed by dynamic light scattering measurements. Figure 6 shows the time-dependent profile of turnover numbers (TONs, mol of H_2_ evolved/mol of **1**) of H_2_ evolution in the reaction of **1** with an excess amount of HCOOH in water at 80 °C. The pH-dependent TON shows a maximum around pH 3.5 (Figure 7). We investigated the dependence of initial rate for H_2_ production against the concentration of **1** (0.63–5.0 mM). This linear correlation clearly indicates that the catalytic reaction is first-order against the catalyst concentration (Figure 8).

**Table 1.** Dehydrogenation of HCOOH, DCOOH, HCOOD, or DCOOD in H_2_O or D_2_O by using **1** as a precursor^a^

Entry	Substrate	Solvent	TON^b^	Ratio of H_2_: HD: D_2_ (%)		
				H_2_	HD	D_2_
1	HCOOH	H_2_O	13.1	100	0	0
2	DCOOH	H_2_O	5.2	7	93	0
3	HCOOD^c^	D_2_O	6.7	1	91	8
4	DCOOD^c^	D_2_O	2.4	0	2	98

^a^ Reaction conditions: **1** (1.25 mM) and HCOOH (2.60 M), DCOOH (2.60 M), or DCOOD (2.60 M) in H_2_O or D_2_O at 80 °C at pH or pD 3.5.

^b^ Turnover number: mol of evolved dihydrogen gases (H_2_, HD, and D_2_)/mol of **1**.

^c^ HCOOD and DCOOD contain 2% H atom.

In order to investigate the reaction mechanism of the dehydrogenation of HCOOH, deuterium isotope-labeling experiments were conducted as shown in Table 1. Entries 2 (DCOOH in H_2_O at pH 3.5) and 3 (HCOOD in D_2_O at pD 3.5) show the TONs of 5.2 and 6.7, respectively, which are lower than that of 13.1 in the entry 1 (HCOOH in H_2_O at pH 3.5). These results suggest that the cleavage of C–H bond in HCOOH and O–H bond in H_2_O should be involved in the rate-determining step. The lowest TON value of 2.4 in entry 4 (DCOOD in D_2_O at pD 3.5) can be expected by the results of entries 2 and 3. According to the results of entries 1–4, we propose the reaction mechanism shown in Figure 9. The bis(μ-formate) complex **1** is converted to the (hydride)(formate) species **2** with the release of CO_2_. The subsequent decarboxylation of **2** is expected to result in formation of a dihydride species **A,** which can be proposed owing to the previously reported analogous structures [20–24]. Then reductive elimination of H_2_ can yield a low-valent species **B**. Protonation of **B** would provide a monohydride species **C**, which can bind HCOO^–^ to return to **2**. This proposed mechanism involves protonation step of **B** and binding step of HCOO^–^, with a p*K*_a_ of 3.75, to **C**, which causes a maximum TON around pH 3.5 because acidic conditions facilitate the protonation step and basic conditions deprotonating HCOOH facilitate the binding step of HCOO^–^ to **C**.

We also investigated the hydrogen isotope exchange reaction by observation of evolved dihydrogen gases in Table 1. The entry 2 shows H_2_ (7%) and HD (93%) are evolved in the reaction of **1** with DCOOH in H_2_O. This result clearly indicates that dihydrogen gases (H_2_ and HD) originate from H of H_2_O and D of DCOOH. The evolved H_2_ is a key product to elucidate the reaction mechanism since H_2_ comes from only H^+^ in H_2_O, which enables us to exclude the possibility of protonation of the hydride ligand to evolve dihydrogen gas. The same is true of entry 3. In this context, we can propose the reaction mechanism of the hydrogen isotope exchange reaction, involving the H^+^/D^+^ exchange of the protic hydride ligand, causing reductive elimination of dihydrogen gas, as shown in Figure 10 [25].

## 4. Conclusions

Isotope-labeling experiments suggest that the formation of dihydrogen gas from formic acid takes place via H^+^/D^+^ exchange of the protic hydride ligands and reductive elimination from the protic hydride ligands of the dinuclear Ru complex in H_2_O/D_2_O.

## Disclosure statement

No potential conflict of interest was reported by the authors.

## Funding

This work was supported by Japan Society for the Promotion of Science (JSPS), [grant numbers JP26000008 (Specially Promoted Research); JP16K05727; JP15K05566]; World Premier International Research Centre Initiative (WPI), Japan.

## Supplementary Material

suppl.zip

## Supplemental data

The supplemental material for this paper is available online at https://doi.org/10.1080/14686996.2017.1379857
