# Supplementary material for: Mechanistic investigation of the formation of H2 from HCOOH with a dinuclear Ru model complex for formate hydrogen lyase
Source: Sci Technol Adv Mater. 2017 Nov 1;18(1):870–6. doi: 10.1080/14686996.2017.1379857 (PMC5678451; doi:10.1080/14686996.2017.1379857)

checkCIF/PLATON report

You have not supplied any structure factors. As a result the full set of tests cannot be run.

THIS REPORT IS FOR GUIDANCE ONLY. IF USED AS PART OF A REVIEW PROCEDURE FOR PUBLICATION, IT SHOULD NOT REPLACE THE EXPERTISE OF AN EXPERIENCED CRYSTALLOGRAPHIC REFEREE.

No syntax errors found.   CIF dictionary   Interpreting this report

Datablock: Ru2_formate_DMSO

```
Bond precision:  O- C = 0.0020 A        Wavelength=0.71075

Cell:            a=16.020(2)     b=7.9505(10)       c=14.045(2)
                 alpha=90        beta=92.996(3)     gamma=90
Temperature:     93 K
```

	Calculated	Reported
Volume	1786.4(4)	1786.4(4)
Space group	P 21/c	P 1 21/c 1
Hall group	-P 2ybc	-P 2ybc
Moiety formula	C10 H14 O10 Ru2 S2	C10 H14 O10 Ru2 S2
Sum formula	C10 H14 O10 Ru2 S2	C10 H14 O10 Ru2 S2
Mr	560.47	560.47
Dx,g cm-3	2.084	2.084
Z	4	4
Mu (mm-1)	1.969	1.969
F000	1096.0	1096.0
F000’	1087.41	
h,k,lmax	20,10,18	20,10,18
Nref	4122	4089
Tmin,Tmax	0.681,0.674	0.511,0.674
Tmin’	0.668	

```
Correction method= # Reported T Limits: Tmin=0.511 Tmax=0.674
AbsCorr = NUMERICAL

Data completeness= 0.992          Theta(max)= 27.549

R(reflections)= 0.0159( 3965)     wR2(reflections)= 0.0512( 4089)

S = 1.359                 Npar= 273
```

The following ALERTS were generated. Each ALERT has the format
test-name_ALERT_alert-type_alert-level.
Click on the hyperlinks for more details of the test.

Alert level C

```
PLAT222_ALERT_3_C Non-Solvent Resd 1   H Uiso(max)/Uiso(min) Range        4.1 Ratio
PLAT391_ALERT_3_C Deviating Methyl C8      H-C-H Bond Angle ......        117 Degree
```

Alert level G

```
CHEMS02_ALERT_1_G  Please check that you have entered the correct
            _publ_requested_category classification of your compound;
            FI or CI or EI for inorganic; FM or CM or EM for metal-organic;
            FO or CO or EO for organic.
            From the CIF: _publ_requested_category   CHOOSE FI FM FO CI CM CO or
            From the CIF: _chemical_formula_sum:C10 H14 O10 Ru2 S2
PLAT164_ALERT_4_G Nr. of Refined C-H H-Atoms in Heavy-Atom Struct.         14 Note
PLAT230_ALERT_2_G Hirshfeld Test Diff for    O5     --  C3      ..        5.5 s.u.
PLAT230_ALERT_2_G Hirshfeld Test Diff for    O6     --  C4      ..        5.1 s.u.
PLAT230_ALERT_2_G Hirshfeld Test Diff for    O7     --  C5      ..        5.3 s.u.
PLAT230_ALERT_2_G Hirshfeld Test Diff for    O8     --  C6      ..        5.4 s.u.
PLAT232_ALERT_2_G Hirshfeld Test Diff (M-X)  Ru1    --  C3      ..       10.6 s.u.
PLAT232_ALERT_2_G Hirshfeld Test Diff (M-X)  Ru1    --  C5      ..        9.7 s.u.
PLAT232_ALERT_2_G Hirshfeld Test Diff (M-X)  Ru2    --  C4      ..       10.3 s.u.
PLAT232_ALERT_2_G Hirshfeld Test Diff (M-X)  Ru2    --  C6      ..       10.9 s.u.
PLAT882_ALERT_1_G Missing datum for _diffrn_reflns_av_unetI/netI .     Please Check
```

---

0 **ALERT level A** = Most likely a serious problem - resolve or explain
0 **ALERT level B** = A potentially serious problem, consider carefully
2 **ALERT level C** = Check. Ensure it is not caused by an omission or oversight
11 **ALERT level G** = General information/check it is not something unexpected

2 ALERT type 1 CIF construction/syntax error, inconsistent or missing data
8 ALERT type 2 Indicator that the structure model may be wrong or deficient
2 ALERT type 3 Indicator that the structure quality may be low
1 ALERT type 4 Improvement, methodology, query or suggestion
0 ALERT type 5 Informative message, check

It is advisable to attempt to resolve as many as possible of the alerts in all categories. Often the minor alerts point to easily fixed oversights, errors and omissions in your CIF or refinement strategy, so attention to these fine details can be worthwhile. In order to resolve some of the more serious problems it may be necessary to carry out additional measurements or structure refinements. However, the purpose of your study may justify the reported deviations and the more serious of these should normally be commented upon in the discussion or experimental section of a paper or in the "special_details" fields of the CIF. checkCIF was carefully designed to identify outliers and unusual parameters, but every test has its limitations and alerts that are not important in a particular case may appear. Conversely, the absence of alerts does not guarantee there are no aspects of the results needing attention. It is up to the individual to critically assess their own results and, if necessary, seek expert advice.

Publication of your CIF in IUCr journals

A basic structural check has been run on your CIF. These basic checks will be run on all CIFs submitted for publication in IUCr journals (*Acta Crystallographica*, *Journal of Applied Crystallography*, *Journal of Synchrotron Radiation*); however, if you intend to submit to *Acta Crystallographica Section C* or *E* or *IUCrData*, you should make sure that full publication checks are run on the final version of your CIF prior to submission.

Publication of your CIF in other journals

Please refer to the *Notes for Authors* of the relevant journal for any special instructions relating to CIF submission.

PLATON version of 27/03/2017; check.def file version of 24/03/2017

Datablock Ru2_formate_DMSO - ellipsoid plot

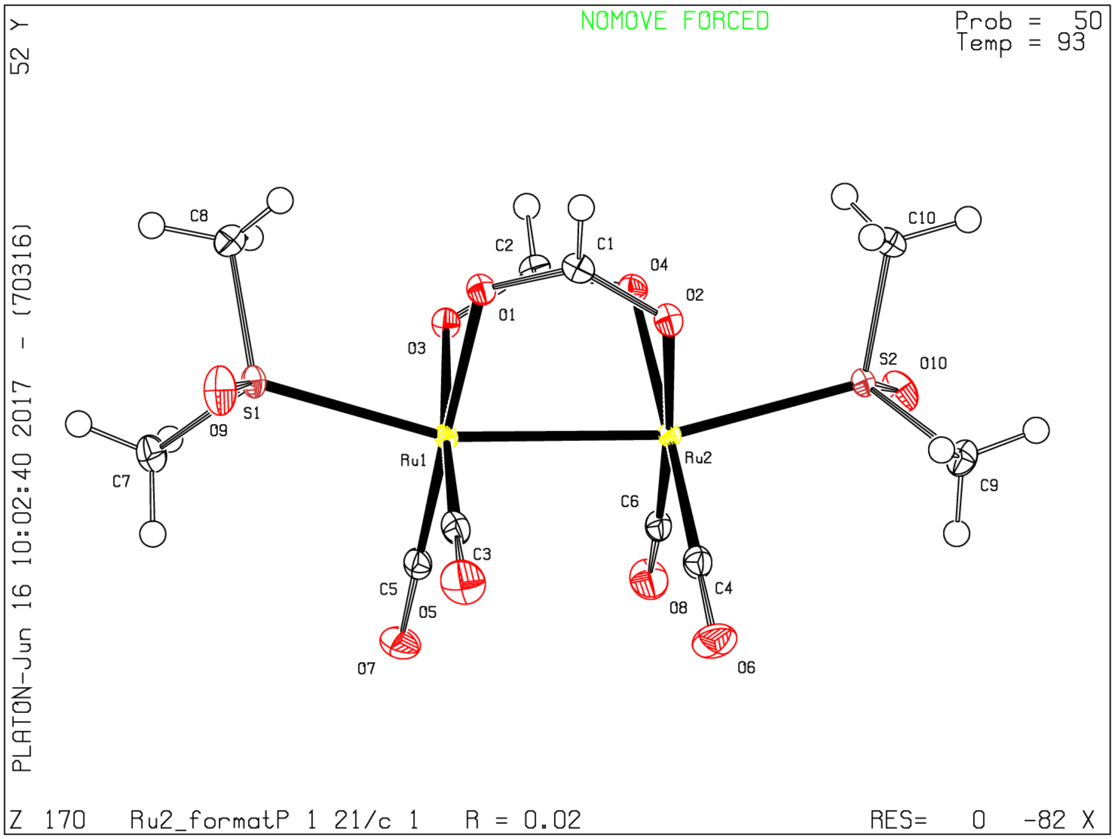

Supplement: suppl.zip [file TSTA_A_1379857_SM9498.zip › suppl1.pdf]
